# Why we need more individual-based parasite research

**DOI:** 10.1186/s13071-026-07567-y

**Published:** 2026-08-11

**Authors:** Gerardo Fracasso, Robert Poulin, Barbara Tschirren

**Affiliations:** 1 Centre for Ecology and Conservation, University of Exeter, Penryn, UK; 2 Department of Zoology, University of Otago, Dunedin, New Zealand

**Keywords:** Parasites, Ectoparasites, Individual-based studies, Population-based studies, Individual variation, Individual-based techniques, Host–parasite interactions, Paradigm shift in parasite-related research

## Abstract

A vast body of literature has investigated the direct and indirect effects of parasites on hosts. While the key role of among-individual variation of host traits has long been acknowledged, individual parasite variation has mostly been overlooked due to the difficulties in studying individual parasites. This has hampered advances in our understanding of fundamental parasite biology as well as progress in the development of novel control strategies. Here, we show how recent technological and methodological innovations, coupled with an improved theoretical understanding, allow for a paradigm shift towards individual-based approaches in parasite-related research and highlight potential breakthroughs across fields. We suggest that ectoparasites—due to their life history as well as medical and veterinary importance—are ideal model systems for the study of individual parasite variation.

**Graphical Abstract:**

**Figure:**
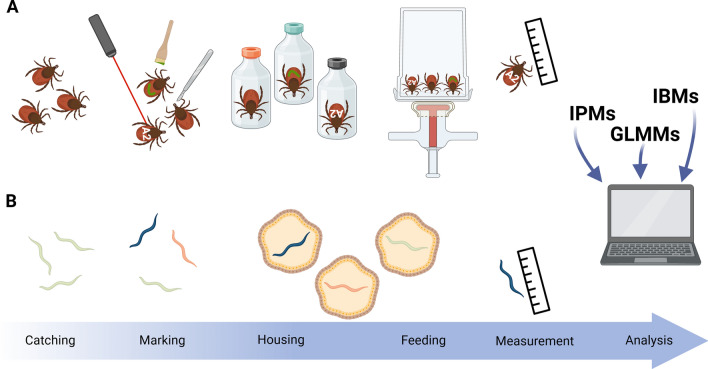

**Supplementary Information:**

The online version contains supplementary material available at 10.1186/s13071-026-07567-y.

## Background

Host–parasite interactions play a fundamental role in shaping the world as we know it [1]. Parasites act as strong selective forces [2], affecting host trait evolution including (but not limited to) genetic diversity [3], morphology, physiology [4], behaviour [5], and life history [6], with significant medical, veterinary, and economic impact worldwide. Consequently, considerable research effort has gone into understanding the direct and indirect effects that parasites have on individual hosts at different spatio-temporal scales and levels of complexity, from host physiological modifications [7] to behavioural effects [8]. Substantial work has also been carried out to investigate how hosts counteract parasites, for example through immunological [9] and behavioural defences [10]. Most of these studies have quantified impacts on individual hosts, and variability among hosts, to better understand repertoires of host responses to parasites. However, unlike research on hosts, much less attention has been devoted to variation among individual parasites [11] hampering our understanding of host–parasite dynamics. Here, we discuss the several drawbacks and limitations given by neglecting inter-individual parasite variation and suggest that ectoparasites are a particularly suitable group where to apply this paradigm shift. We present a number of recent theoretical, methodological and technological advances that allow to overcome the challenges traditionally associated with the study of individual parasites.

## Beyond populations: why individual parasites matter

To date, the vast majority of **parasite-related research** (see Glossary for a glossary of terms in bold) has relied on population-level approaches. These are relatively quick to set up and run (no individual marking and tracking required) and for a number of species and research questions they might still be the best, or only, viable option. Yet, they have several notable drawbacks and limitations with respect to individual-based approaches that should thoroughly be considered during the study design. For example, population-level studies cannot incorporate inter-individual variation in morphology (e.g., body size), physiology (e.g., thermal tolerance, metabolic rate), behaviour (e.g., **host preference**) or life history (e.g., ageing, previous hosts) which prevents addressing fundamental and still open questions in parasite biology (see section “Parasites as individuals: examples and applications”) as well as the development of more effective control measures. Furthermore, population-level data have statistical caveats and limited versatility (see below). Individual-based approaches permit us to fill these knowledge gaps (Table 1 for a succinct comparison). Indeed, recent studies using individual-based approaches in (non-parasitic) invertebrates have provided key insights into, for example, cognition, personality, physiology and microbiome composition, and their consequences for an individual’s fitness [12, 13]. Yet this approach is only recently starting to be used on parasites [14] with relatively few species investigated and a limited body of empirical evidence. Indeed, evidence on intraspecific variation in ectoparasites (especially mites) is starting to accumulate, for instance on parasite susceptibility to infection [15], host preference [16], attachment propensity [17] and phenotypic plasticity [18]. From the genetic standpoint, sometimes clonal parasite or pathogen lineages can be obtained allowing the study of genotype-by-genotype (G × G) host–parasite interactions as well as genotype-by-environment interactions (G × E). This approach is becoming increasingly common [19–21]. Furthermore, the characterisation of inter-individual variation through **-omics** approaches is revolutionising several fields, including microbiology that traditionally focused on population-level processes [22, 23].

**Table 1:** Characteristics of population-based and individual-based approaches

Approach		
	Population based	Individual based
Lifetime fitness	Only possible for semelparous species	**Iteroparous** and **semelparous** species
Age	Often impossible or poorly accurate. Where possible, estimated indirectly from biomarkers	Directly estimated in all cases
Social network	Not possible	Measurable
Resource distribution and allocation	Partially possible if different genotypes can be associated to different allocation strategies	Measurable
Phenotypic plasticity	Changes in population means and variances. Individual plasticity not measurable (often lower than the population range)	Changes in population and individual means and variances
Repeatability	Only for population averages	Measurable
Interstage effects	Population level effects. Prone to ecological fallacy	Measurable
Intergenerational effects	Population level effects. Prone to ecological fallacy	Measurable
Behaviour	General patterns. Prone to ecological fallacy	Animal personalities and behavioural syndromes
Physiology	General patterns. Prone to ecological fallacy	Within-individual dynamics
Cost and workload	Relatively low	High
Versatility	Low	High

Comparison of measurable traits and effects between population-based and individual-based approaches on parasite-related research

A crucial advantage of individual-based approaches is that they allow measuring the same individual multiple times (longitudinal studies), and thus to calculate **repeatability** of traits of interest. If kinship relationships between individuals are known, the proportion of individual variation that is heritable can furthermore be calculated both in the parasite [24] and in the host [25]. In animal behaviour, repeatable measures over time and across contexts are defined as personalities and have also been documented in invertebrate taxa [26]. Parasite personalities can have ecological and evolutionary consequences similar to those of non-parasitic species [27] but with additional parasite-specific effects such as, for instance, on host burden following host attachment preference [17] but have so far received very little attention. Moreover, longitudinal studies permit us to investigate lifetime reproductive success and the strength of selection acting on traits of interest, which would not be possible in population studies.

Individual approaches allow to measure inter-individual variation in host preference and host-seeking strategies, and their cascading effects on parasite survival [28, 29], mate choice [30], lifetime reproductive success [31], and vector-borne disease (VBD) dynamics. Also, the role of microbiome composition in modifying parasite physiology and behaviour can be investigated [4, 14], including the effects of **vector manipulation** and previous feeding events. Another advantage is the capability to quantify **phenotypic plasticity** in response to environmental change [32], to detect carry-over effects within- and between life stages [33], and inter-generational effects such as whether individual behaviour or physiology is affected by parental age or parental host choice. Lastly, the ecological significance of intraspecific variation can be estimated [34].

Beyond elucidating fundamental evolutionary, ecological, and parasitological dynamics, insights into among-individual variation can improve current parasite control strategies and help develop novel, more effective and sustainable management interventions. This has important implications for both human and animal health, as well as significant economic repercussions. Finally, individual-based studies often collect multiple data types and tissue samples. Besides giving a better understanding of an individual status these can be analysed from multiple perspectives, thus making the study more time- and cost-effective, and adherent to the principles of Replacement, Reduction and Refinement (3Rs) by allowing us to answer multiple scientific questions with the same dataset, thereby preventing the need to carry out additional experiments. This reduces the number of animals used for scientific purposes.

We wish to point out that the concept of individual-based parasite studies is clearly not new and that experiments have been carried out on individual parasites over the past decades (see for example [35–37]). Yet, such studies are the exception rather than the norm and they mostly focused on a single time point in a parasite’s life cycle. We argue that, in contrast to vertebrate species, the benefits of individual-based approaches have been underappreciated in parasite-related research and wish to redress this balance.

## A role for ectoparasites

Individual-level research on endoparasites, such as helminths, has now become possible thanks to modern technologies ranging from sophisticated culture mediums [38] to the use of organoids to grow parasites in vitro [39]. However, among all parasites, **ectoparasites** offer unique opportunities for individual-level studies. Ectoparasites such as mosquitoes, ticks, mites, fleas, flies, and lice are medically relevant as they transmit several VBDs such as malaria, Dengue, Zika, Lyme, and Chagas, to name a few. Yearly, these account for millions of cases and more than 700 000 deaths [40] with their incidence being on the rise due to anthropogenic climate change, urbanisation and globalisation [41]. Livestock, companion animals, and crops are also affected by VBDs transmitted by ectoparasites such as, for example, mange, babesiosis or trypanosomiasis [42, 43] causing substantial economic burden worldwide, especially in developing countries [1]. Insights gained from individual-based studies on ectoparasites are thus likely to have noteworthy benefits across the One Health framework [44].

Ectoparasites feed externally on the host and, when off it, they can be found in the surrounding environment thus making it easy to follow them throughout their life cycle. Moreover, many species such as mosquitoes and ticks are sufficiently large to be individually marked, manipulated, and monitored using both established and new technologies (see below). Additionally, rearing in semi-natural conditions is often feasible, allowing year-round maintenance [45]. Host–ectoparasite interactions are also easier to measure and to experimentally manipulate than endoparasites. Studying ectoparasites often requires less invasive methods which has practical and ethical benefits. Finally, many ectoparasites are pathogen vectors and are thus excellent models to study the multi-way interactions between the host, the parasite, and the pathogen.

**Fig. 1:**
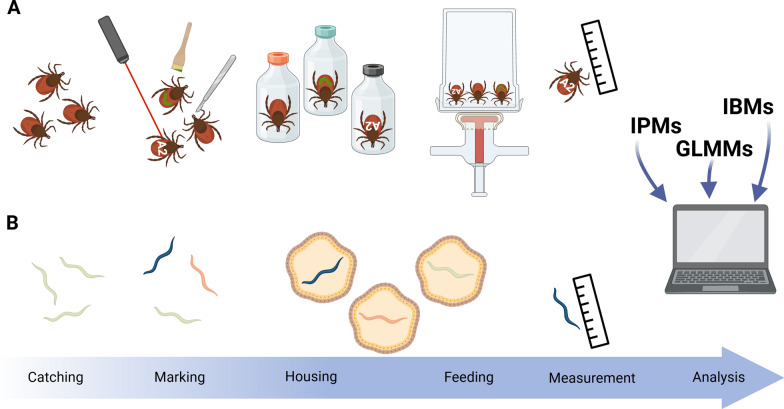
An individual-based workflow. A representative workflow for an individual-based study of ectoparasites (**A**) and endoparasites (**B**). For endoparasites, housing and feeding can occur at the same time using organoids or mediums in vials. Created in Biorender (https://BioRender.com/dxdz1f1)

Until recently, methodological challenges—perhaps compounded by an attitude that failed to fully acknowledge parasites as individuals in their own right—have hampered individual-based parasite-related research. Yet, novel techniques have recently been developed that, in combination with new statistical tools, facilitate the study of individual parasite variation both in field and laboratory conditions throughout a parasite’s life cycle (Fig. 1). Furthermore, decades of individual-based research on free-living vertebrates provide a solid theoretical framework. We advocate that time is ripe for a paradigm shift in parasite-related research from a population-level approach to an individual-level one.

## Available techniques

### Marking and tracking

Most ectoparasites are arthropods with relatively small bodies, few or no flat surfaces, and a chitinous exoskeleton [46]. Additionally, some species extensively swell their integument during feeding and go through moulting shortly afterwards (e.g., hard ticks). In these species marking can be challenging as these characteristics prevent the use of many traditional marking techniques. Moreover, the chosen technique should be quick and inexpensive. Luckily, solutions have been developed to cope with these issues such as (fluorescent) dyes, powders and paints [47, 48], laser or other engraving technique [49], clipping [24] or by embedding molecular compounds in the animal’s body [50] (Additional file 1: Table S1 for a list of tools and methods for individual-based studies). If parasites are sufficiently large (e.g., salmon louse and other copepods) **RFID tags** can also be used [51]. Notably, some of these techniques are also suitable for endoparasites, e.g., fluorescent dyes [52]. New marking and tracking techniques allow to investigate several individual behaviours such as mate choice, host preference, tactic responses to environmental cues, dispersal, and to repeatedly measure traits (capture–mark–recapture). Among new markers, fluorophores are highly visible under UV light, cost-effective, persistent, non-invasive and easy to apply [53]. For ectoparasites whose body swells dramatically, limb clipping has proven to be effective for individual recognition with limbs often regrowing at moulting [24]. This technique can generally be applied even to the smallest life stages.

Another important recent advancement is tracking technology. The ever-increasing computational power and machine learning tools enable tracking of individuals in contexts and ways that were unimaginable only a few years ago [54]. Recently developed tracking software such as AnimalTA [55] are free, easy to use, and can track multiple individuals at the same time across a range of conditions. Machine learning tools are starting to be used for automatic image recognition (computer vision) of parasites and disease signs [56], and to individually monitor or track small invertebrates [57] with enormous potential for both fundamental and applied science. Additionally, they can integrate motion information not only providing individual tracking but also classifying what an animal is doing [58]. Although currently mainly used to identify animals at the group or species level [59], these tools have potential to be used for individual recognition as well [60]. Moreover, a handful of automated video-tracking software has been recently developed that record individual flight paths up to a few metres at high temporal and spatial resolution both in the lab and in the field ([61] for a review). For studies at ranges up to dozens of metres, radio and harmonic tags fitted on the animal can be detected by radar and lidar technologies to track individual paths [62]. The smallest tags weigh just a few milligrams and allow to track arthropods in the wild [63]. Given the current pace of growth in computational power and advances in device miniaturisation, further breakthroughs in individual parasite tracking are envisaged.

It goes beyond the scope of this article to discuss the characteristics and limitations of every technique and technology in detail (see [64] for a review of techniques) but the best method will depend on the needs of the study and particularly: (i) on the species and life stage to mark (e.g., size, shape, weight, body swelling, exposure to water or oils); (ii) the required marking duration; and (iii) whether the mark should be passed on to the next life stage.

### Laboratory rearing and feeding systems

Numerous ectoparasite species exhibit low survival rates at every life stage with a minority of individuals reaching reproductive maturity. Oftentimes, life cycles are tied to seasonal conditions such as temperature, humidity or host availability. Moreover, carrying out individual-based studies might additionally require individuals with very specific characteristics which might be difficult to catch in sufficient numbers in the wild. The establishment of lab populations can be instrumental to solve these difficulties. For instance, population size and demographic parameters can be easily manipulated in laboratory populations to obtain enough individuals with certain characteristics (e.g., life stage, sex). In fact, conditions such as temperature, humidity, brightness, and food availability can be manipulated thus altering the life cycle speed. Indeed, laboratory populations of mosquitoes and ticks have been established [65, 66]. However, we argue that the considerable potential of laboratory populations, in conjunction with permanent individual marking techniques for individual-based research, has remained largely untapped. The same is true for the many existing laboratory cultures of helminths [67]. We think the available expertise in marking and laboratory rearing already provide a solid knowledge base awaiting to be exploited for individual-based studies.

Understanding the dynamics and mechanisms of host–parasite interactions during ectoparasite feeding has historically been challenging. To simplify and standardise feeding interactions, promising artificial feeding systems have been developed. Artificial feeding is a powerful and versatile technique where the host blood and integument properties are simulated [68]. This technique has several benefits and has been successfully used on several species of hematophagous ectoparasites (e.g., mosquitoes, ticks, Tsetse-flies, sandflies, bedbugs). First, host characteristics are simulated in a standardised way thus removing the confounding effects of host variability. Therefore, individually marked ectoparasites can be artificially fed to disentangle the individual effects caused by the vertebrate host’s traits (e.g., blood properties) from those associated with parasite characteristics. Second, besides its use for ectoparasite rearing, artificial feeding can be effectively used to test repellent compounds or host preference, and to extract vector compounds (e.g. salivary extracts) [68]. Third, individual (or clonal colonies of) pathogens can be safely administered to the parasite to investigate pathogen effects on individual vectors while minimising concerns related to biosafety and vertebrate host welfare.

Many ectoparasitic species can feed on multiple hosts within a life stage, and it can be hypothesised that previous feeding events have carry-over effects on, for instance, the parasite microbiome and behaviour which in turn have implications for disease transmission and surveillance. Yet, no such prior information is available at first catch from the wild. Fortunately, recent molecular tools allow to determine the host on which the parasite had its last blood meal [69], even when mixed blood meals occurred [70], and even identify host individuals [71].

### The -omics realm

Until recently, the physiological condition and processes occurring within an individual could only be inferred from few biomarkers. However, as with all proxies based on one or few markers, care needs to be taken when drawing conclusions about the more general picture. The development of -omics techniques provides opportunities for a much more detailed analysis of the physiology of individuals and its regulation by looking at whole pathways rather than single markers. In fact, it is now possible to study the genome of an individual, how it is transcribed into RNA and translated into proteins, which metabolic pathways get activated in response of that (and to what extent) as well as the role of host-associated microbial communities in these processes [23]. -Omics approaches have high temporal resolution making them ideal to study responses to abiotic and biotic stressors [72]. It has thus become viable to study host–parasite–pathogen interactions at an unparalleled level of detail [73, 74]. Importantly, the advent of -omics has amplified the potential of individual studies by allowing a fine-scale analysis of internal processes for all entities of the interaction: the host, the parasite, and the pathogen.

### The final step: statistical analysis

Accounting for individual variation in the statistical analysis is essential to avoid loss of information. Moreover, failure to account for among-individual variation can lead to interpretation fallacies. Two examples are **ecological fallacy** [75] and **Simpson’s paradox** [76]. In both cases, statistical relationships among variables within groups (e.g., among individuals) are wrongly inferred or concealed when data are grouped. Furthermore, in case of non-linear relationships between variables—which are common in biological systems—the average value taken from a population will be an under- or over-estimation respect to the value of the average individual (Jensen’s inequality) thus negatively affecting estimates and predictions [77]. Furthermore, it is harder (if not impossible) to account for confounding variables such as the environment experienced by individuals or individual variation in resource acquisition and allocation [78].

Until a few decades ago, the computational power needed to run models that could account for individual variation was a limiting factor. Now, a range of powerful and versatile software enable to include individual identities. General(ized) Linear Mixed Models (GLMMs) are a prime example of this [79]. These models allow accounting for differences in an individual’s trait mean and in the rate of its change, by fitting different intercepts and slopes for each individual while simultaneously accounting for other effects [80]. Additionally, random effects provide information on the amount of variance explained by a given factor such as how much individual parasite identity affects model results. Also, random effect residuals from LMMs can be used for further analyses such as testing their correlation with other behavioural and ecological variables, although with some caveats [81]. Furthermore, the versatility of (G)LMMs allows to study two or more response variables at once, as well as their co-variation (multivariate models). Finally, random effects can accommodate the kinship relationship among individuals to estimate trait **heritability** [82]. Both the host and parasite genetic relatedness matrix could be fitted simultaneously to disentangle their respective effects [83]. (G)LMMs also allow to estimate within-individual repeatability through various methods (reviewed in [84]) as, for instance, the intraclass correlation coefficient (ICC) or the predictability and its effect size [84].

When the aim is modelling and predicting population dynamics over time, two statistical approaches permit researchers to incorporate among-individual variation: individual-based models (IBMs) and integral projection models (IPMs). IBMs stochastically simulate how a discrete number of individuals behave and interact with each other and the environment based on fitness-related decision rules [85]. A set of rules and occurrence probabilities is defined along with the number of iterations to produce population-level outcomes over different time scales. To date, IBMs have been used to test theoretical scenarios and study emergent (population) properties from individual behaviours, e.g., variation in disease transmission following behavioural changes [86]. They can incorporate heterogeneity at spatial, ontogenetic, behavioural, cognitive, phenotypic and genetic levels [87]. A drawback of IBMs is that it can be difficult to get a statistical summary of the results (but see [88]), and if applied to specific cases they require exhaustive knowledge of the species and its ecology. IPMs instead focus on describing and predicting how populations change over time particularly when individual traits vary continuously (e.g. size, age). A structured population model is built using continuous individual-state variables to describe variation in the individual vital rates underlying changes in demography and population growth [89].

Finally, the strength of artificial intelligence foundation models (of which large language models are a subset) in learning generalisable representations of reality has sparked interest in their biological applications, including in epidemiology [90]. These tools have a great and still mostly unexplored potential in giving predictions and finding patterns with limited data. Yet, the rules underlying such models might not always be biologically meaningful or grounded, with model responses prone to **hallucinations**. Although hallucinations might not be entirely avoidable with the current model architectures, they can be significantly reduced by setting appropriate reasoning rules and through broad knowledge integration [91, 92]. In this context, individual-level data could help to provide solid mechanistic rules on which to train increasingly accurate models.

## Parasites as individuals: examples and applications

The adoption of individual-based approaches in parasite research has the potential to address several unresolved questions across diverse research domains. In this section, we present examples of key outstanding questions that may be tackled across disciplines and discuss how insights gained can be translated into practical applications and applied research contexts.

### Outstanding questions

#### Life history and condition

- Are there different life-history strategies within a parasite population?
- What is the impact of age and energy reserves on parasite performance?
- What carry-over effects occur across life stages and generations?

#### Host choice and exploitation

- Is an individual host of equal value to different parasites within a species? Is there a preference for hosts in poor or good condition based on parasite condition or genotype?
- Do past feeding events influence future host choice and exploitation?
- Is there individual variation in host preference and host-seeking strategies?
- Which factors regulate the area and timing of host attachment and detachment in hematophagous ectoparasites?
- Does intraspecific morphological variation (e.g., to the feeding apparatus) affect host choice and exploitation? How?

#### Selection and phenotypic plasticity

- Do parasites intra-specifically differ in their capability to exploit different hosts, and ultimately in their fitness?
- Which parasite traits are under selection? How can we mitigate evolutionary responses to antiparasitic treatments?
- What are the ecological and evolutionary implications of developmental plasticity in parasites?
- How does climate change affect phenotypic plasticity, host preference, and host-seeking behaviour?

#### Parasite microbiome and behaviour

- How does individual microbiome composition affect parasite behaviour and physiology?
- Do parasites show learning capabilities or individual-level variation in tactic (taxis) responses?
- What are the ecological and evolutionary consequences of parasite personalities? How do they affect host attachment and disease transmission?

### Practical examples

#### Phenotypic plasticity

Parasites can differ intra-specifically in plasticity [18]. Hence, if individual ectoparasites differ, for instance, in thermal and water-loss tolerance they will experience different heatwave consequences with non-plastic individuals having lower survival. Understanding how much plasticity parasites can express and what proportion of the population can express a determined level of trait plasticity will improve predictions on population dynamics with respect to environmental change.

#### Life-history strategies

Parasites might have either high virulence, fecundity and low survival or low virulence and fecundity but longer lifespan. Depending on which parasitic individuals exploit a host their virulence (and thus the effect on host fitness) will differ. Moreover, parasite populations made up by multiple strategies might be more resilient to fluctuating environmental conditions.

#### Lifetime reproductive success

Measuring individual fitness permits researchers to determine how selective pressures shape parasite traits. For example, if morphological differences in feeding organs are favoured in some mosquito populations and disadvantageous in others due to host compatibility.

#### Personality, host preference and seeking behaviour

Parasites can prefer hosts based on a wide range of traits or characteristics including their reproductive status [93], sex [28] developmental stage [94], body size [16], infection status [95], or metabolic rate [96]. Such differences in host preference and host-seeking behaviour might lead to different feeding likelihoods on a host species, sex or age group. This will have consequences for VBD transmission as different hosts might differ in pathogen competence. Similarly, for parasites using their hosts as carriers, parasite dispersal might be affected as different hosts might disperse differently.

#### Infection status

If parasites infected with a specific pathogen are more likely to parasitise a certain group of hosts (vector manipulation) this might change parasite burden for the hosts (affecting host condition) as well as the probability to be infected with the pathogen itself. A deeper knowledge of the causes and consequences of vector manipulation would help to understand how parasite populations and VBDs are maintained in the wild, informing control strategies.

### Applied research

A paradigm shift towards individual-level studies could help address the growing concern around the evolution of resistance to antiparasitics. If an individual approach is used, -omics techniques would allow to identify new drug targets [97] while the potential for resistance evolution (trait heritability) could be measured, and inform parasite control measures accordingly. For instance, by targeting old individuals as selection acts less strongly at older age [98].

To determine exposure risk in a region, great effort is made to predict parasite and pathogen distribution and abundance. Both are dependent on the response of parasites (and of the vectored pathogens) to changing environmental conditions (phenotypic plasticity). Measuring among-individual variation in plasticity would improve predictive models of species distribution helping to target disease prevention and mitigation strategies for humans, livestock, companion and wild animals. This is particularly crucial in the context of climatic change.

Interestingly, some parasites can exploit such a wide range of host species that it is questioned whether they are composed by multiple specialised sub-species [99]. Understanding such within-population variation is key to inform parasite control policies and improve our predictions of disease transmission as some host–pathogen routes become more likely than others. Individual-based approaches can address such question by quantifying individual host preference and its associated fitness.

## Conclusions

We argue that time is ripe for a paradigm shift towards individual-based studies in parasite-related research thanks to recent methodological advancements. Using examples from other taxonomic groups where individual-based approaches are commonplace, we highlighted the numerous benefits that this approach could provide over more traditional population-based approaches, and which outstanding questions could be addressed. Crucially, individual-based approaches can prompt further questions on the origin and maintenance of among-individual variation (e.g. environmental vs. genetic), and how this affects evolutionary trajectories [100]. We discussed how newly developed tools can help achieving such paradigm shift at all stages of a study.

A few characteristics of individual-based studies might hinder this paradigm shift. Economic uncertainty (exacerbated by limited long-term funding options) as well as political and environmental instability are threats that should be accounted for. This is particularly true for long-term studies in developing countries where parasite impacts are often high. However, the major obstacles might come from prevailing practices and mindsets that overlook the importance of intraspecific variation among parasites. We hope this article provided food for thought.

To date, tools and methods allow to easily track parent–offspring relationships in laboratory populations. In the future, new techniques could allow to expand such tracking in the wild. This advancement would pave the way for long-term individual-based parasitological studies similar to those in free-living vertebrates. Long-term studies are invaluable tools with numerous practical and theoretical benefits [101]. For instance, both inter-generational effects and selection events can be repeatedly measured in a range of conditions. Furthermore, the scientific value of long-term studies increases over time. In fact, the ever-growing sample size permits more complex statistical analyses. Additionally, new molecular, statistical and computational advancements often allow analyses that would not have been possible at the time of sample collection, similarly to the value of museum samples [102].

## Supplementary Information

Additional file 1.

## Data Availability

Data supporting the main conclusions of this study are included in the manuscript.

## Glossary

Ecological fallacy: A statistical phenomenon when a relationship observed at the group level is incorrectly assumed to hold at the individual level
Ectoparasite: A parasite (see definition below) that feeds on the host surface either temporarily or permanently
Hallucinations: An artificial intelligence model response which is factually incorrect, misleading, or entirely made up which is presented confidently as if it was true
Heritability: The proportion of phenotypic variation (variance) in a trait within a population which is due to (additive) genetic variation rather than environmental variation
Host preference: The innate or learned tendency to exploit a host species or group of individuals more than others when all hosts are equally available. Preference can be expressed at different stages of the host-parasite interaction and have consequences for individual parasite fitness
Iteroparous: Reproductive strategy where an organism can reproduce multiple times throughout its life cycle
Life-history strategies: Describes how an organism balances key biological processes (e.g., age at maturity, number and size of offspring, reproductive frequency, or lifespan) to maximize its reproductive success (fitness)
-Omics: An umbrella term including genomics (DNA), transcriptomics (RNA), proteomics (proteins), lipidomics (lipids), metabolomics (metabolites), epigenomics (epigenetic marks), and metagenomics (microbiome)
Parasite-related research: Studies and research lines that make use of parasites to answer questions. It does not exclusively refer to parasitology but includes all research fields investigating host-parasite interactions
Phenotypic plasticity: Potential of a single genotype to produce a range of different phenotypes in response to environmental conditions
Repeatability: The proportion of variance attributable to differences between individuals
RFID tags: Small (minimum 1.25 × 7 mm) and light (0.03 g) devices which use Radio Frequency Identification technology (RFID). Also called Passive Integrated Transponder (PIT) tags they contain unique 10–15 digit alphanumeric ID codes that are put on an individual and are read by an external antenna
Semelparous: Reproductive strategy where an organism can reproduce only once throughout its life cycle
Simpson’s paradox: Related to ecological fallacy (but not the same) it occurs when a relationship between two variables appears between separate groups of data but reverses or disappears when the groups are combined
Vector manipulation (adaptive): Alterations caused by a microorganism to the physiology or behaviour of its vector (e.g., ticks, mosquitoes) in a way that enhances the microorganism’s probability of transmission and survival
