# Supplementary material for: Why we need more individual-based parasite research
Source: Parasit Vectors. 2026 Aug 11;19:330. doi: 10.1186/s13071-026-07567-y (PMC13463528; doi:10.1186/s13071-026-07567-y)
Supplement: Supplementary file 1 [file 13071_2026_7567_MOESM1_ESM.docx]

**Table S1. Tools and methods for individual-based studies.**

| Research step | Available tools and software |
| --- | --- |
| Marking | Dyes, fluorophores, powders, paints, enamels, laser engraving, limb clipping, RFID tags, isotopes, immunomarkers, photoluminescent tags |
| Tracking | AnimalTA^i^, BehaveAI^ii^, Insect detect^iii^, BugBox^iv^, EthoVision XT^v^, Braid^vi^, Motus^vii^, OpenCV^viii^, Photonic Fence^ix^, StreamPix^x^, Trackit3D^xi^, Hyperspectral lidar, infrared detector |
| Feeding | Animal/human models, artificial membrane feeding, capillary feeding, engineered biocompatible materials (resembling host skin) |
| Molecular analysis | Genomics, transcriptomics, proteomics, lipidomics, metabolomics, epigenomics, metagenomics |
| Statistical analysis | - Frequentist GLMMs: lme4^xii^, lmerTest^xiii^, glmmTMB^xiv^, nlme^xv^, MASS^xvi^, spam^xvii^, GLMMadaptive^xviii^, coxme^xix^ - Bayesian GLMMs: brms^xx^, MCMCglmm^xxi^, rstanarm^xxii^, INLA^xxiii^, rjags^xxiv^, RStan^xxv^ - IBMs: SpaDES^xxvi^, NetLogoR^xxvii^, ABM^xxviii^, Rcpp^xxix^, simecol^xxx^ - IPMs: IPMpack^xxxi^, ipmr^xxxii^, lefko3^xxxiii^ |

A non-comprehensive list of available tools and methods to help carrying out individual-based studies. The “Statistical analysis” row refers to available R packages.

# Resources

^i^ Chiara, V. and Kim, S.-Y. (2023) AnimalTA: a highly flexible and easy-to-use program for tracking and analysing animal movement in different environments. *Methods in Ecology and Evolution* 14, 1699–1707. <https://doi.org/10.1111/2041-210X.14115>

^ii^ Troscianko, J. *et al.* (2026) BehaveAI enables rapid detection and classification of objects and behavior from motion. *PLoS Biol.* 24, e3003632. 10.1371/journal.pbio.3003632

^iii^ Sittinger, M. *et al.* (2024) Insect detect: an open-source DIY camera trap for automated insect monitoring. *PloS One* 19, e0295474. <https://doi.org/10.1371/journal.pone.0295474>

^iv^ Welch, K.D. and Lundgren, J.G. (2024) Introducing BugBox: a platform for AI-assisted bioinventories of arthropods. *American Entomologist*, 70(3), 31-33. <https://doi.org/10.1093/ae/tmae047>

^v^ Noldus, L.P. *et al.* (2001) EthoVision: A versatile video tracking system for automation of behavioral experiments. Behav. Res. Methods Instrum. Comput. 33(3), 398–414. <https://doi.org/10.3758/bf03195394>

^vi^ Straw Lab. Braid: multi‑camera acquisition and tracking software. Available from: <https://strawlab.org/braid/>

^vii^ Taylor, P.D. *et al.* (2017) The Motus Wildlife Tracking System: A collaborative research network to enhance the understanding of wildlife movement. *Avian Conservation & Ecology*, 12(1). <https://doi.org/10.5751/ACE-00953-120108>

^viii^ Bradski, G. (2000) The OpenCV Library. Dr. Dobb’s Journal of Software Tools, 120; 122-125.

^ix^ Hyde, R.A. *et al.* (2018) *Photonic Fence*. U.S. Patent Application 20180089504.

^x^ NorPix, *StreamPix* (RRID:SCR_015773) [Software for digital video recording]. <https://www.norpix.com/products/streampix/streampix.php>

^xi^ Revopoint 3D Technologies Inc. TrackIt 3D Optical Tracking Scanner. Available from: <https://www.revopoint3d.com/pages/trackit-optical-tracking-3d-scanner>

^xii^ Bates, D. *et al.* (2015) Fitting linear mixed-effects models using lme4. *Journal of Statistical Software*, 67(1), 1-48. <https://doi.org/10.18637/jss.v067.i01>

^xiii^ Kuznetsova A *et al.* (2017). “lmerTest Package: tests in linear mixed effects models.” *Journal of Statistical Software*, 82(13), 1-26. <https://doi.org/10.18637/jss.v082.i13>

^xiv^ Brooks, M.E. *et al.* (2017) glmmTMB balances speed and flexibility among packages for zero-inflated generalized linear mixed modeling. *The R Journal*, 9(2), 378-400. <https://doi.org/10.32614/RJ-2017-066>

^xv^ Pinheiro J *et al.* (2025) nlme: linear and nonlinear mixed effects models. <https://doi.org/10.32614/>

^xvi^ Venables, W.N. and Ripley, B.D. (2002) Modern applied statistics with S. Fourth Edition. Springer, New York. ISBN 0-387-95457-0

^xvii^ Furrer R. and Sain S.R. (2010) spam: a sparse matrix R package with emphasis on MCMC methods for gaussian markov random fields. *Journal of Statistical Software*, 36(10), 1–25. <https://doi.org/10.18637/jss.v036.i10>

^xviii^ Rizopoulos, D. (2025) *GLMMadaptive: generalized linear mixed models using adaptive gaussian quadrature*. R package version 0.9-7. <https://github.com/drizopoulos/GLMMadaptive>

^xix^ Therneau, T.M. (2024) *coxme: mixed effects cox models*. [https://CRAN.R-project.org/package=coxme](https://cran.r-project.org/package=coxme)

^xx^ Bürkner, P.C. (2021) Bayesian item response modeling in R with brms and Stan. *Journal of Statistical Software*, 100(5), 1-54. <https://doi.org/10.18637/jss.v100.i05>

^xxi^ Hadfield, J.D. (2010) MCMC methods for multi-response generalized linear mixed models: the MCMCglmm R package.” *Journal of Statistical Software*, **33**(2), 1–22. <https://www.jstatsoft.org/v33/i02/>

^xxii^ Goodrich B *et al.* (2025) rstanarm: Bayesian applied regression modeling via Stan. <https://mc-stan.org/rstanarm>

^xxiii^ Rue, H. *et al.* (2009) Approximate Bayesian inference for latent Gaussian models by using integrated nested Laplace approximations. *Journal of the Royal Statistical Society Series B: Statistical Methodology*, *71*(2), 319-392. <https://doi.org/10.1111/j.1467-9868.2008.00700.x>

^xxiv^ Plummer, M. (2025) *rjags: Bayesian Graphical Models using MCMC*. [https://CRAN.R-project.org/package=rjags](https://cran.r-project.org/package=rjags)

^xxv^ Stan Development Team (2025) RStan: the R interface to Stan. <https://mc-stan.org/>

^xxvi^ Chubaty, A. and McIntire E. (2024) *SpaDES: Develop and Run Spatially Explicit Discrete Event Simulation Models*. [https://spades.predictiveecology.org](https://spades.predictiveecology.org/)

^xxvii^ Bauduin, S *et al.* (2025) *NetLogoR: build and run spatially explicit agent-based models*. [https://CRAN.R-project.org/package=NetLogoR](https://cran.r-project.org/package=NetLogoR)

^xxviii^ Ma, J (2025) *ABM: agent based model simulation framework*. <https://github.com/junlingm/abm>

^xxix^ Eddelbuettel, D. *et al.* (2026) *Rcpp: seamless R and C++ integration*.  [https://doi.org/10.32614/CRAN.package.Rcpp](%20https://doi.org/10.32614/CRAN.package.Rcpp)

^xxx^ Petzoldt, T. and Rinke, K. (2007) Simecol: an object-oriented framework for ecological modeling in R. *Journal of Statistical Software*, *22*, 1-31. <https://doi.org/10.18637/jss.v022.i09>

^xxxi^ Metcalf, C.J.E. *et al.* (2013) IPM pack: an R package for integral projection models. *Methods in Ecology and Evolution*, 4(2), 195-200. <https://doi.org/10.1111/2041-210x.12001>

^xxxii^ Levin, S *et al.* (2021). ipmr: flexible implementation of integral projection models in R.” *Methods in Ecology and Evolution*, 12. <https://doi.org/10.1111/2041-210X.13683>

^xxxiii^ Shefferson, R *et al.* (2021) lefko3: analyzing individual history through size-classified matrix population models. *Methods in Ecology and Evolution*, 12(2), 378-382. <https://doi.org/10.1111/2041-210X.13526>
